# Impact of a Functional Intervention with Home Follow-Up on Respiratory Symptoms and Functionality in Oncology Patients: A Randomised Clinical Trial

**DOI:** 10.1007/s00520-026-10725-8

**Published:** 2026-04-29

**Authors:** Eduado J. Fernandez-Rodriguez, Celia Sanchez-Gomez, Emilio Fonseca-Sanchez, Maria Isabel Rihuete-Galve, Juan Jesús Cruz Hernández

**Affiliations:** 1 Department of Nursing and Physiotherapy, University of Salamanca, Salamanca, Spain; 2 Department of Developmental and Educational Psychology, University of Salamanca, Salamanca, Spain; 3 Department of Medicine, University of Salamanca, Salamanca, Spain; 4 Medical Oncology Service, University Hospital of Salamanca, Salamanca, Spain; 5 Salamanca Biomedical Research Institute (IBSAL), Salamanca, Spain

**Keywords:** Dyspnea, Neoplasms, Occupational therapy, Caregiver burden, Activities of daily living, Home care services

## Abstract

**Purpose:**

This study aimed to determine whether an Effort Re-education Programme (ERP) delivered after hospital discharge yields greater improvements in functionality than Conventional Clinical Practice (CCP) in oncology patients with associated respiratory symptoms.

**Methods:**

A stratified randomised clinical trial was conducted including 65 oncology patients recruited during hospitalisation and followed after discharge. Participants were allocated to either CCP or CCP plus a home-based functional Effort Re-education Programme. Functionality (Barthel Index) was the primary outcome. Secondary outcomes included dyspnoea severity (mMRC), general performance status (ECOG), and caregiver burden (Zarit scale). Assessments were performed at discharge (baseline), 15 days, and one month post-discharge.

**Results:**

Patients receiving ERP showed significantly greater improvements in functionality compared with the control group (mean change: + 20.3 vs. + 6.6 points; p < 0.001). Significant between-group differences were also observed for dyspnoea (p = 0.002), performance status (p < 0.001), and caregiver burden (p < 0.001). No hospital readmissions were recorded in the intervention group during follow-up. Length of hospital stay was shorter in the intervention group prior to discharge.

**Conclusion:**

A home-based Effort Re-education Programme initiated at hospital discharge significantly improves functional outcomes, respiratory symptoms, and caregiver burden in oncology patients, supporting its integration into discharge planning and continuity-of-care models.

The clinical trial was registered in ClinicalTrials.gov (NCT06035263). Registration Date: 2023–11-01; 04:11 h.

**Supplementary Information:**

The online version contains supplementary material available at 10.1007/s00520-026-10725-8.

## Background

Recent oncological advances have improved survival and led to the emergence of long-term survivors [1], but cumulative treatments frequently impair quality of life through symptoms such as fatigue, anxiety, and dyspnoea [2–4], which is especially prevalent in advanced disease, affecting up to 41% of palliative care patients and more than 73% of lung cancer patients [5, 6]. Dyspnoea is often perceived as an uncontrollable limitation, prompting avoidance behaviours that increase inactivity and reduce functional capacity [7], generating fear-avoidance patterns similar to those observed in chronic pain, chronic fatigue syndrome, and fibromyalgia [8, 9], and contributing to the “respiratory patient cycle” of deconditioning and effort-related dyspnoea [10]. As conventional pharmacological treatments are frequently insufficient for managing multifactorial dyspnoea [11, 12], comprehensive re-adaptive interventions are necessary to prevent passive adaptation, physical deterioration, progressive disability, and negative impacts on mobility and psychosocial well-being [13].

These findings indicate that associated respiratory conditions are a frequent yet undervalued problem in oncology practice [14], often hindering patients’ return to normal life due to clinical deterioration or difficulties in transferring hospital-based learning to daily routines. The National Comprehensive Cancer Network (NCCN) highlights the importance of educational strategies and energy conservation techniques within comprehensive functional rehabilitation programmes for cancer patients with respiratory symptoms [11], supported by a multicentre trial of 296 patients showing reduced dyspnoea and fatigue-related symptom burden following programme implementation [14–16]. These strategies, including activity pacing, task prioritisation and planning, scheduled rest, posture optimisation, simplification of daily activities, and use of assistive devices, aim to reduce exertional dyspnoea and prevent inactivity-related functional decline, and their effectiveness is further supported by a meta-analysis of 113 studies involving 11,525 patients, which demonstrated significant improvement with non-pharmacological interventions (pooled effect size 0.30; 95% CI, 0.25–0.36; p < 0.001) [17].

Non-pharmacological psychosocial interventions are effective in managing these symptoms, sometimes surpassing pharmacological treatments, which supports the use of a biopsychosocial and multidisciplinary approach [18–22]. It is also important to address fear-avoidance factors such as kinesiophobia, defined as an excessive fear of movement that may lead to inactivity and functional decline in oncology patients with respiratory involvement. This can be assessed using validated tools such as the modified Tampa Scale of Kinesiophobia [23, 24].

The selection of the most appropriate intervention setting should be based on clinical complexity and patients’ self-management capacity, supporting the implementation of a supervised, individualized home-based programme after hospital discharge to improve access and adherence [25, 26]. Evidence shows that supervised remote follow-up and structured programmes lead to better physical activity outcomes and adherence [27–29]. In cancer patients, multimodal exercise interventions combining aerobic and strength training, together with functional re-education and health education adapted to individual capacities, achieve the best results [10, 21, 30–33]. Accordingly, this study proposes an interdisciplinary intervention involving occupational therapists, nurses, and medical specialists to enhance conventional care through functional re-education and environmental adaptation following discharge.

Study Objectives:

The primary objective of this study was to evaluate whether a home-based Effort Re-education Programme produces greater improvements in activities of daily living than Conventional Clinical Practice in oncology patients with associated respiratory symptoms.

Secondary objectives were to assess changes in dyspnoea severity, general performance status, and caregiver burden, and to explore associations between baseline clinical characteristics and functional outcomes.

## Materials and Methods

### Design

This research was a stratified, prospective, longitudinal, randomised controlled trial with a fixed parallel assignment, comprising an experimental group and a control group. The study was conducted in a mixed setting: specialised care at the University Hospital Complex of Salamanca (CAUSA), specifically within the Medical Oncology Service, and at the University of Salamanca, within the Teaching and Assistance Unit of Occupational Therapy (UDATO). This randomised controlled trial was reported in accordance with the CONSORT 2010 guidelines. The CONSORT checklist is provided as Supplementary Material.

### Participants

Eligible participants were adult oncology patients hospitalised for cancer-related complications who presented with associated respiratory symptoms and moderate to severe functional dependency at discharge. Participants were selected through consecutive sampling based on the following criteria:

Inclusion Criteria: Adult oncology patients (≥ 18 years) recruited during hospitalisation in the Medical Oncology Service of the University Hospital of Salamanca, with a histopathological diagnosis of cancer (newly diagnosed or recurrent), who presented with associated respiratory symptoms and moderate to severe functional dependency at the time of hospital discharge, defined by a Barthel Index score between 20 and 55 points.

Additional inclusion criteria were clinical stability at discharge and the ability to participate in a home-based functional intervention programme. All participants were required to provide written informed consent.

Exclusion Criteria: Patients with moderate to severe cognitive impairment (Mini-Mental State Examination score < 24); severe anaemia (haemoglobin < 10 g/dL); terminal disease with an expected survival of less than one month; or medical contraindications preventing participation in functional re-education or home-based follow-up.

Withdrawal criteria: Withdrawal criteria included patient death, disease progression leading to a terminal condition during follow-up, hospital readmission at the time of scheduled home visits, or incomplete final assessment.

### Sample Size Calculation

The sample size calculation was based on the study’s primary variable, the Barthel Index, considering a minimally clinically important difference of 4.8 points. This estimate was derived from a pilot study conducted in an oncology population with similar characteristics, which observed a standard deviation of 9.7 points.

To detect this difference with a statistical power of 80% and a two-sided significance level of α = 0.05, it was determined that 32 participants per group were required. Assuming a 10% risk of loss to follow-up, the sample size was increased accordingly. The calculation was performed using the Epidat 4.2 software.

### Randomisation and Blinding

Participants were randomly allocated to the intervention or control group using a computer-generated random sequence (1:1 ratio) created by an independent researcher. Allocation concealment was ensured until assignment. Outcome assessors and data analysts were blinded to group allocation.

Blinding was ensured at multiple levels. Allocation and sequencing were managed by personnel not involved in the intervention or evaluation. Participants were unaware of their assigned group and the corresponding intervention. To avoid bias, evaluations were conducted by trained external researchers who were blinded to group assignments. Additionally, data analysts were also blinded to group allocation to ensure scientific rigour.

### Procedures and Data Collection

Participants were recruited during hospitalisation in the Medical Oncology Service of the University Hospital of Salamanca (CAUSA), where eligible patients were identified by the oncology team and screened by the research staff. Recruitment occurred after clinical stabilisation and before discharge, and all participants provided written informed consent following receipt of verbal and written study information prior to baseline assessment and randomisation.

Evaluation Timeline:

Evaluations were conducted at three time points:

- Baseline evaluation at hospital discharge (after inclusion, before randomisation).
- Follow-up evaluation at 15 days.
- Final evaluation one month after the baseline assessment.​

**Fig. 1:**
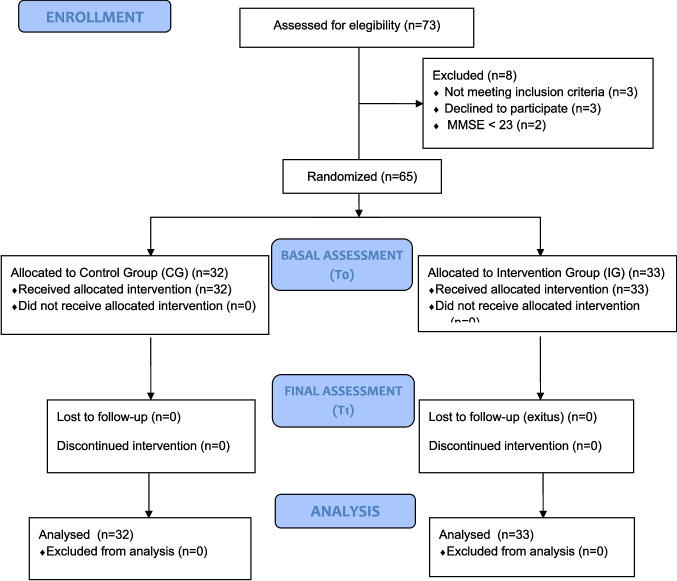
CONSORT flow diagram of participants included on the trial

Each evaluation employed the same tools. Baseline data included demographic, clinical, and outcome variables. Evaluations were performed by trained research personnel. The study flow diagram is presented in Fig. 1.​

Result Reporting.

Participants may request an individualised report with the results of their evaluations.​

### Outcomes

#### Primary Outcome

Activities of Daily Living (ADLs): Assessed using the Barthel Index validated in Spanish. Scores range from 0 (total dependence) to 100 (total independence) [34].​

#### Secondary Outcomes

- Respiratory pathology/dyspnoea: Assessed using the modified Medical Research Council (mMRC) dyspnoea scale, which classifies dyspnoea severity into five levels [35].
- Health-related quality of life (HRQoL): Measured with the Eastern Cooperative Oncology Group (ECOG) Performance Status, ranging from 0 (fully active) to 5 (deceased) [36].
- Caregiver burden: Measured with the short version of the Zarit Burden Interview, a 7-item Likert-type questionnaire. Scores above 17 suggest significant caregiver burden [37].

### Interventions

Two parallel intervention programmes were designed for the two study groups. The first group, the Control Group (CG), received Conventional Clinical Practice (CCP), which included pharmacological treatment and a Health Education Programme. The second group, the Intervention Group (IG), received the control group's intervention plus a Programme for Effort Re-education (PER). All interventions were developed in accordance with the TIDieR checklist, detailed in Supplementary Material 1.​

Both programmes were structured and supervised by the research team from the University of Salamanca (Spain).​

A. Control Group (CG): Conventional Clinical Practice: Pharmacological Treatment + Health Education Programme.

Participants received a health education programme through a dossier provided at discharge, containing information on the importance of physical activity, nutrition, and hydration to maintain a healthy lifestyle.​

B. Intervention Group (IG): Programme for Effort Re-education (PER) to Improve Performance in Activities of Daily Living.

The Effort Re-education Programme commenced at hospital discharge and continued for one month in the patient’s home environment. The intervention consisted of structured sessions delivered by trained occupational therapists, with one session per week (approximately 60 min each).

Sessions included functional re-education in activities of daily living, training in energy conservation techniques, breathing control strategies, environmental adaptation, and prescription of assistive devices when necessary. Intervention fidelity and adherence were monitored during each visit.

This included the interventions described for the control group and the following additional components:​

1. Functional Re-education.

Initiated before discharge and continued at home, including:​

- Direct intervention in ADLs.
- Energy conservation techniques (ECTs).
- Sleep hygiene advice based on NCCN guidelines.​

2. Prescription of Assistive Products and Environmental Adaptations.

An evaluation before discharge and at home to recommend assistive products and identify environmental barriers to independence.​

The intervention commenced at hospital discharge and continued at home for one month.​

The home-based component of the ERP consisted of structured, supervised follow-up visits by trained professionals, who monitored adherence, corrected activity performance, and adapted the programme to each patient’s functional and environmental context. Outcome assessments were carried out during these scheduled visits.

### Visit Schedule

Each participant attended three evaluation visits:​

Baseline visit: before discharge, including complete data collection and randomisation.

Follow-up visits (15 days and 1 month): conducted at UDATO. Structure similar to the baseline visit, excluding sociodemographic data.

Each visit lasted approximately one hour.​ Assessments were conducted at discharge (baseline), 15 days, and one month post-discharge during scheduled follow-up visits.

## Data Analysis

The statistical analysis plan was established a priori, prior to study initiation. Any modifications made after data collection were limited to data cleaning procedures, verification of eligibility criteria, and correction of potential data entry errors, without altering the predefined analytical strategy or outcome comparisons.

Statistical analyses were conducted according to the intention-to-treat principle. The normality of the variables was assessed using the Kolmogorov–Smirnov test (p < 0.05) and by visual inspection of histograms. A descriptive analysis was subsequently performed, with quantitative variables reported as medians and interquartile ranges, and qualitative variables as frequencies and percentages.

For inferential analysis, between-group comparisons were carried out using the Wilcoxon test. The magnitude of the intervention effect was estimated by calculating Cohen’s d effect sizes, interpreted as small (0.2), medium (0.5), and large (0.8). Associations between study variables and questionnaire scores were examined using Spearman’s rank correlation coefficient. Statistical significance was defined using a two-sided 95% confidence interval, with p-values < 0.05 considered statistically significant. All analyses were performed using IBM SPSS Statistics (version 28.0.1).

## Results

### Baseline Sociodemographic and Clinical Characteristics

Data from 65 patients were analysed, including 32 in the control group and 33 in the experimental group. The groups were comparable in terms of age and sex distribution, with a mean age of 64.6 ± 15.0 years in the control group and 66.2 ± 12.2 years in the experimental group; men accounted for 59.4% and 63.6% of participants, respectively. Inferential analyses confirmed the absence of statistically significant differences in baseline sociodemographic and clinical characteristics.

Lung cancer was the most frequent histopathological diagnosis in both groups (43.8% in the control group and 45.5% in the experimental group), followed by breast, colorectal, and prostate cancer. Metastatic disease was more prevalent in the control group (81.3%) than in the experimental group (75.0%). During the follow-up period, 37.5% of patients in the control group experienced at least one hospital readmission, whereas no readmissions were recorded in the experimental group. These characteristics are summarised in Table 1.

**Table 1:** Demographic and clinical data baseline assessment

VARIABLES			IG (N = 33)	CG (N = 32)
Age			66.24 (± 12,15)	64.59 (± 14.9)
Sex		Male	63.6%	59.4%
		Female	36.4%	40.6%
Pathological diagnosis		Breast	21.2%	9.4%
		Lung	45.5%	43.8%
		Digestive system	6.1%	9.4%
		Pancreas	6.1%	6.3%
		Prostate	3%	9.4%
		Other	18.2%	18.8%
Number of oncology treatment lines			3 (1)	3 (4)
Barthel			54.09 (± 20.05)	49.53 (± 22.44)
ECOG			3 (2)	2 (2)
MRC			2.33 (± 1.13)	2.41 (± 1.07)
ZARIT			64.39 (± 16.68)	70.62 (± 21.40)
Hospitalized time			11 days	12 days
Metastasis	Yes		75.8%	81.2%
	No		24.2%	18.8%
Hospital readmission	Yes		3%	37.5%
	No		97%	62.5%

### Clinical and Functional Variables

The mean number of treatment lines was 3.00 (SD = 0.92) in the control group and 3.21 (SD = 0.96) in the experimental group.

Regarding functionality as measured by the Barthel Index, a progressive improvement was observed in both groups, more pronounced in the experimental group. In this group, the mean score increased from 54.09 (SD = 20.06) to 74.38 (SD = 20.66), while in the control group it rose from 49.53 (SD = 22.45) to 56.13 (SD = 21.59).

Dyspnoea, measured by the MRC scale, showed a decrease in both groups. In the experimental group, the mean score decreased from 2.33 (SD = 1.14) to 1.16 (SD = 0.99), and in the control group from 2.41 (SD = 1.01) to 1.68 (SD = 1.08).

The ECOG scale reflected an improvement in functional status across the three time points assessed. In the experimental group, the score decreased from 2.30 (SD = 0.88) to 1.25 (SD = 0.88); in the control group, from 2.00 (SD = 0.80) to 1.84 (SD = 0.73).

Finally, caregiver burden as measured by the Zarit scale decreased more markedly in the experimental group (from 64.39 to 44.78) than in the control group, where it remained practically unchanged (from 70.62 to 69.81).

The mean number of hospitalisation days was slightly lower in the experimental group (11.41 days; SD = 3.41) compared to the control group (12.22 days; SD = 3.11). All these data are presented in Tables 1 and 2.

**Table 2:** Within-group changes and between-group comparisons for primary and secondary outcomes

Variables	Intervention group (IG) (n = 33)		Control Group (CG) (n = 32)		P value	Cohen´s d
	Baseline assessment (T0)	Final Assessment (T2)	Baseline assessment (T0)	Final Assessment (T2)		
Barthel	54.09 (± 20.06)	74.38 (± 20.66)	49.53 (± 22.45)	56.13 (± 21.59)	< 0.001	0.680
MRC	2.33 (± 1.14)	1,16 (± 0.99)	2,41 (± 1.01)	1.68 (± 1.08)	0.002	0.620
ECOG	2.30 (± 0.88)	1,25 (± 0.88)	2,00 (± 0.80)	1.84 (± 0.73)	< 0.001	1.310
ZARIT	64.39	44.78	70.62	69.81	< 0.001	1.010

### Comparative Analysis Between Groups

A paired sample test was conducted to compare outcomes between the experimental and control groups across the main clinical and functional variables. The analyses revealed statistically significant differences in all variables studied, favouring the experimental group. Change scores were calculated as the difference between post-intervention and baseline values, with positive values indicating improvement for the Barthel Index and negative values indicating improvement for mMRC, ECOG, and Zarit scores.

In terms of general functional status measured by the ECOG index, a significant improvement was observed in the experimental group compared to the control (t(31) = 7.41; p < 0.001), with a mean difference of 0.97 points (95% CI: 0.70–1.24). The effect size was large (Cohen’s d = 1.31; Hedges’ g = 1.28).

Functionality in basic activities of daily living, assessed using the Barthel Index, also improved significantly in the experimental group, with a mean difference of 12.66 points (95% CI: 5.96–19.35; t(31) = 3.85; p < 0.001). The effect size was moderate (Cohen’s d = 0.68; Hedges’ g = 0.67).

Regarding the degree of dyspnoea, measured by the MRC scale, a significant reduction was observed in the experimental group compared to the control group (mean difference = 0.56; 95% CI: 0.23–0.89; t(31) = 3.48; p = 0.002), with a moderate effect size (Cohen’s d = 0.62; Hedges’ g = 0.60).

Finally, caregiver burden assessed with the Zarit scale showed a notable and significant difference in favour of the experimental group, with a mean reduction of 21.38 points (95% CI: 13.78–28.97; t(31) = 5.74; p < 0.001), with a large effect size (Cohen’s d = 1.01; Hedges’ g = 0.99). All these results can be seen in Table 3.

**Table 3:** Between-group comparison of changes in primary and secondary outcomes from baseline to one-month follow-up

Outcome measure	Control group (Mean change)	Experimental group (Mean change)	Between-group difference	p-value
Barthel Index (functionality)	+ 6.6	+ 20.3	+ 13.7	< 0.001
Dyspnoea (mMRC)	− 0.3	− 1.1	− 0.8	0.002
Performance status (ECOG)	− 0.4	− 1.2	− 0.8	< 0.001
Caregiver burden (Zarit scale)	− 2.1	− 8.5	− 6.4	< 0.001

### Correlation Analysis

Exploratory analyses were conducted to examine whether baseline clinical characteristics were associated with changes in functional and symptomatic outcomes over time.

A positive and significant correlation was observed between functional status measured by the ECOG scale and dyspnoea (MRC) (r = 0.400; p < 0.001), as well as with caregiver burden (Zarit) (r = 0.311; p = 0.012). Conversely, the correlation between ECOG and the Barthel Index was negative (r = −0.534; p < 0.001).

The MRC scale also showed positive correlations with the Zarit scale (r = 0.474; p < 0.001) and negative correlations with the Barthel Index (r = −0.619; p < 0.001), reflecting that greater dyspnoea is associated with higher caregiver burden and lower functionality.

Caregiver burden (Zarit) showed a strong negative correlation with the Barthel Index (r = −0.628; p < 0.001), and a positive correlation with age (r = 0.391; p = 0.001) and number of hospitalisation days (r = 0.294; p = 0.018).

Age also showed a significant negative correlation with functionality (Barthel) (r = −0.268; p = 0.031), and the number of hospitalisation days correlated significantly with the number of treatment lines (r = 0.342; p = 0.006).

No significant correlations were found between ECOG and age (r = 0.206; p = 0.100) or between MRC and age (r = 0.132; p = 0.296).

## Discussion

This study evaluated the effectiveness of an Effort Re-education Programme (ERP) compared to Conventional Clinical Practice (CCP) in improving functionality in activities of daily living among hospitalised oncology patients with associated respiratory conditions. The findings demonstrate that the ERP yields significant clinical benefits, including improvements in functional ability (Barthel Index), reduction in dyspnoea (mMRC scale), better general performance status (ECOG), reduced caregiver burden (Zarit scale), and shorter hospital stays. These findings directly address the study’s primary research question and provide additional evidence regarding changes in dyspnoea, general functional status, and caregiver burden.

The improvement in functionality, reflected by an average increase of 20 points in the Barthel Index in the experimental group, confirms the utility of the ERP in restoring patient autonomy within a hospital setting. This improvement was significantly greater than that observed in the control group, which showed a mean increase of 6.6 points in the Barthel Index compared with a 20.3-point increase in the intervention group and aligns with the findings of Henke et al. (2014) [38], who reported similar effects in patients with advanced lung cancer undergoing strength and endurance training programmes.

Dyspnoea, one of the most debilitating symptoms in oncology patients with respiratory involvement, also showed a significant reduction in the experimental group. This finding is consistent with the results reported by Güneş et al. (2020) [39], who demonstrated that home-based breathing and relaxation exercises improved both functional capacity and perception of respiratory symptoms. The improvement observed in the mMRC scale in our study further underscores the value of incorporating breathing control techniques into inpatient rehabilitation programmes.

General performance status, measured via the ECOG scale, showed an average reduction of over one point in the experimental group, indicating better effort tolerance and less physical deterioration. Similar favourable outcomes have been reported in prior studies on physical interventions for patients with advanced-stage cancer [22–25].

One of the most significant contributions of this study is the demonstration that the ERP positively impacts caregiver burden. In our study, the Zarit scale score significantly decreased in the experimental group, whereas it remained virtually unchanged in the control group. This finding confirms that clinical improvements in patients can have a beneficial effect on their family environment, reducing the emotional and physical load of the primary caregiver. Studies such as that of Ning et al. (2021) [40], which implemented structured continuous care, reported similar outcomes regarding reduced caregiver fatigue and burden.

The correlation analysis identified key relationships between functionality, general performance status, dyspnoea, and caregiver burden. It was observed that greater functional impairment (ECOG) was associated with increased dyspnoea (mMRC) and greater caregiver burden (Zarit), while better functionality (Barthel) correlated with reduced dyspnoea and lower caregiver burden. These associations address the fourth objective of the study and reinforce the need for a holistic approach encompassing both physical and psychosocial dimensions in cancer care.

Additionally, the experimental group recorded no hospital readmissions during the follow-up period and showed a reduction in the average length of hospital stay compared to the control group. Although these outcomes were not primary endpoints, they suggest potential improvements in healthcare system efficiency through functionality-oriented and self-management-based interventions.

Our findings are in line with a recent systematic review by Leyns et al. (2024) [41], which identified exercise as one of the most effective non-pharmacological interventions for addressing the symptom cluster of dyspnoea, fatigue, and physical dysfunction in patients with lung cancer. Likewise, studies on digital interventions, such as those by Huang et al. (2019) [42], have shown improvements in quality of life and symptom distress, while approaches like Acceptance and Commitment Therapy (Li et al., 2022) [43] have reported additional emotional and psychological benefits. Although our programme did not include digital or psychotherapeutic components, the results suggest that structured physical intervention may have cross-cutting effects across various domains of well-being.

The study by Edbrooke et al. (2019) [44], which implemented a multidisciplinary home-based programme, showed that even when objective improvements in functional tests such as the six-minute walk test are not observed, patients report better perceived quality of life and symptom relief. In this regard, the functional and symptomatic benefits observed in our study reinforce the importance of combining objective and subjective measures to assess the real impact of interventions.

In this regard, the functional and symptomatic benefits observed in our study reinforce the importance of combining objective measures, such as the Barthel Index and ECOG Performance Status, with subjective measures, including perceived dyspnoea (mMRC scale) and caregiver burden (Zarit scale), to comprehensively assess the real impact of interventions in oncology care.

### Limitations

This study presents certain limitations. The sample size was relatively small, which may limit the generalisability of the findings. Additionally, the follow-up period was restricted to hospitalisation, so medium- and long-term effects were not assessed. Specific measures of quality of life and the patient’s psychological dimensions were not included, aspects that could be considered in future research to assess the overall impact of the ERP.

## Conclusion

The results of this study demonstrate that the Effort Re-education Programme is an effective strategy for improving functionality, reducing dyspnoea, enhancing general performance status, and lowering caregiver burden in oncology patients with respiratory involvement. Moreover, the associations found between functionality, symptoms, and caregiver burden underscore the need for comprehensive and interdisciplinary care. These findings support the integration of structured functional interventions into hospital care programmes, with the aim of providing more person-centred, efficient, and humanised care.

## Supplementary Information

Below is the link to the electronic supplementary material.

Supplementary file1 (DOCX 20 KB)

## Data Availability

Upon study completion, data will be shared through FAIRsharing on re3data.org.

## Declarations

### Trial Registration and Scientific Rigor

This study follows the SPIRIT 2013 guidelines for protocol writing and the CONSORT 2010 guidelines for reporting randomised controlled trials.​
