# Supplementary material for: Impact of a Functional Intervention with Home Follow-Up on Respiratory Symptoms and Functionality in Oncology Patients: A Randomised Clinical Trial
Source: Support Care Cancer. 2026 Apr 29;34(5):477. doi: 10.1007/s00520-026-10725-8 (PMC13128706; doi:10.1007/s00520-026-10725-8)
Supplement: Supplementary file 1 — Supplementary file1 (DOCX 20 KB) [file 520_2026_10725_MOESM1_ESM.docx]

**Supplementary Material 1. Interventions According to the TIDieR Guidelines:**

Two parallel intervention programmes were designed for the two study groups. The first group received Conventional Clinical Practice (CCP), which included pharmacological treatment and a Health Education Programme. The second group received the control group intervention in addition to an Effort Re-education Programme (ERP), in accordance with TIDieR guidelines.

Both programmes were structured and supervised by the research team from the University of Salamanca (Spain).

**A. Control Group (CG): Conventional Clinical Practice – Pharmacological Treatment + Health Education Programme**

**Short name:** Health Education Programme

**Rationale:** Upon hospital admission, the medical team prescribed pharmacological treatment based solely on the patient's symptoms. The nursing team provided instructions and recommendations to maintain a healthy lifestyle, promoting self-care and good care practices as part of a health education programme. The programme focused on the benefits of an active lifestyle and general guidance to follow.

**What (materials):** Necessary medications were used for symptom control, and health education programme recommendations were delivered via an informative dossier.

**What (procedures):** Instructions and recommendations were provided to promote self-care and good care practices as part of a health education programme aimed at maintaining a healthy lifestyle. Participants received a dossier containing the programme’s instructions and recommendations.

**Who provided the intervention:** The interventions were delivered by a team member with professional experience and qualifications.

**How:** The material was provided individually to each patient upon admission.

**Where:** All sessions were conducted at the University Hospital Complex of Salamanca (CAUSA).

**When and how much:** The intervention was administered daily during the hospital stay, and participants were informed of the schedule and duration.

**Tailoring:** Adaptations were considered. Given the range of topics and the individual nature of the intervention, the sessions were tailored to each subject.

**How well (planned):** Therapy was supervised through weekly meetings between therapists and researchers. Sessions were conducted twice weekly.

**B. Intervention Group (IG): Effort Re-education Programme (ERP) to Improve Performance in Activities of Daily Living**

**Short name:** Effort Re-education Programme (ERP)

**Rationale:** This is a non-pharmacological, interdisciplinary intervention that integrates occupational therapy, nursing, and oncology. Its aim is to address the needs of oncology patients by providing training in knowledge and strategies for correctly carrying out activities of daily living, thus promoting autonomy and maximum functionality. The intervention is based on the principle of complementing and integrating both disciplines.

**What (materials):** Necessary materials included mobility aids such as walking frames, canes or wheelchairs; support products to preserve the integrity of patients' tissues, such as pressure-relief cushions, heel protectors, wedges and other positioning devices; and support products for daily activities such as dressing, personal hygiene and eating, in addition to an incentive spirometer.

**What (procedures):**
**Effort Re-education Programme:** The intervention consisted of effort re-education, delivered by an interdisciplinary team of occupational therapists and nursing staff. The following components were included:

- **Progressive mobilisation:**
  An individual assessment was carried out, in which assistive products were prescribed to facilitate patient mobility (e.g., a walking frame adapted for oxygen therapy, portable oxygen concentrator for daily activities).
  A daily record of patient activities was maintained and adjusted according to clinical condition. This enabled the establishment of a suitable routine to enhance autonomy by making full use of the patient's capabilities without exceeding them. This record was completed by the patient and supervised by the occupational therapist.
- **Grading and Simplification of Activities of Daily Living: Teaching of Energy Conservation Techniques:**
  Individual training was provided on rules for simplifying activities. The training included:
  ‐ Careful organisation of workspaces and adaptation of activity plans.
  ‐ Placement of objects within the patient’s reach.
  ‐ Prioritisation of basic self-care activities in a seated position.
  ‐ Movement control, promoting slow, coordinated, and smooth displacements, avoiding impulsive or vigorous movements.
  ‐ Alternation of strenuous and light activities, with appropriate pauses between them.
  ‐ Balancing periods of activity with periods of rest.

In addition to this intervention, participants in the intervention group also received the same components as the control group, including pharmacological treatment and the Health Education Programme.

**Who provided the intervention:** All interventions were carried out by occupational therapists, nursing staff, and specialist physicians experienced in oncology care.

**How:** The intervention was delivered on an individual basis.

**Where:** Sessions were held in person and individually in the Inpatient Unit of the Medical Oncology Department at the University Hospital Complex of Salamanca, Spain.

**When and how much:** Each participant received one individual session per day during their hospital stay. Each session lasted one hour.

**Tailoring:** Sessions were individually adapted for each patient according to complexity, due to the clinical diversity and the personalised nature of the intervention.

**How well (planned):** The intervention programme was planned and supervised through weekly meetings with all members of the research team. Two sessions were held per week, and strict attendance monitoring was implemented.

| **Component** | **Control Group (CG): Conventional Clinical Practice** | **Intervention Group (IG): Effort Re-education Programme (ERP)** |
| --- | --- | --- |
| **What (materials)** | Necessary medications for symptom control; informative dossier with health education recommendations. | Mobility aids (e.g., walking frames, canes, wheelchairs); tissue integrity support products (e.g., pressure-relief cushions, heel protectors); support products for daily activities (e.g., dressing, hygiene, eating); incentive spirometer. |
| **What (procedures)** | Instructions and recommendations to promote self-care and good care practices for a healthy lifestyle. | Progressive mobilisation; daily activity recording; grading and simplification of daily living activities; energy conservation training. |
| **Who provided the intervention** | Team member with professional experience and qualifications. | Occupational therapists, nursing staff, and specialist physicians. |
| **How** | Material delivered individually to each patient upon admission. | Intervention delivered individually to each patient. |
| **Where** | University Hospital Complex of Salamanca (CAUSA), Spain. | University Hospital Complex of Salamanca, Medical Oncology Department, Spain. |
| **When and how much** | Daily during hospital stay, with information provided on the schedule and duration. | One individual session per day during hospital stay, lasting one hour. |
| **Tailoring** | Sessions adapted to the individual, considering the range of topics and the personalised nature of the intervention. | Sessions individually tailored based on clinical condition and complexity. |
| **How well (planned)** | Supervised through weekly meetings with therapists and researchers. Sessions twice per week. | Program supervised through weekly team meetings. Sessions held twice per week, with strict attendance control. |
| **Rationale** | Pharmacological treatment and health education to promote a healthy lifestyle. | Interdisciplinary approach to improve autonomy and functionality in daily activities. |
